# A Comparison of Commercially Available Digital Microscopes for Their Use in Bench-Model Simulation of Microsurgery

**DOI:** 10.1055/s-0044-1787980

**Published:** 2024-07-11

**Authors:** Laura Awad, Zakee Abdi, Benjamin J. Langridge, Akul Karoshi, Peter E. M. Butler

**Affiliations:** 1 Charles Wolfson Centre for Reconstructive Surgery, Royal Free Hospital, London, United Kingdom; 2 Department of Plastic Surgery, Royal Free Hospital, London, United Kingdom; 3 Division of Surgery and Interventional Science, University College London, London, United Kingdom

**Keywords:** microsurgery simulation, microscope, remote learning, surgical simulation

## Abstract

**Background** Surgical education has seen a transition in the delivery of training, with increased use of online platforms to facilitate remote learning. Simulation training can increase access to education and reduce cost implications, while reducing patient risk. This study aims to compare commercially available digital microscopes, alongside a standard binocular surgical microscope, and determine whether they can be used as an alternative tool for remote microsurgery simulation.

**Methods** Data were collected for a total of four microscopes, including three commercially available digital microscopes, smartphone, and a binocular table microscope. Product characteristics were collated, and a subjective assessment was conducted using an 11-criteria questionnaire, graded with a 5-point scale. Results of digital microscopes were compared with the table binocular microscope.

The Kruskal–Wallis test was used to compare the performance of digital microscopes to the standard binocular microscope

**Results** The questionnaire was completed by 31 participants: two consultants, nine surgical registrars, fourteen junior trainees, and six medical students. Digital microscopes were found to be significantly more affordable and convenient for trainees; however, the cost of the smartphone was significant. Overall, the Pancellant Digital Microscope performed the poorest, with trainees commenting on its unsuitability for surgical practice; the Plugable USB Digital Microscope (PLDM) was rated overall most like the binocular table microscope. The Depth of field was shallow in all digital microscopes.

**Conclusion** With the increasing role of remote learning and simulation training in surgical education, the PLDM can provide a cheaper, more accessible alternative for junior trainees, in their pursuit of microsurgical skill acquisition.

Microsurgery is a surgical discipline that combines the use of operating microscopes with specialized precision tools, using complex surgical techniques to conduct microvascular, microneural, and microlymphatic repairs.

While technological advances are being made in this field, such as augmented reality and fluorescence imaging, current clinical practice utilizes optical binocular microscopes to facilitate surgery. 1 The binocular microscope is undoubtedly a critical tool and facilitates innovative applications across a multitude of surgical specialties including plastic surgery, neurosurgery, and ophthalmology. Since the introduction of the operating microscope by Nylén in 1921, the instrument has undergone significant revisions to improve its performance in this field. 2

Key requirements of the operating microscope include an adequate view of the surgical field, adjustable magnification for varying structures, and bright illumination of the field, with minimal reflection to optimize vision. 1 Additionally, these tools have been adapted to improve surgical positioning, mechanical design efficiency, and recording/display abilities.

Optical microscopes have some disadvantages including high cost, difficult transportation due to their size, and relative inaccessibility for trainees, which present obstacles and limitations to surgical education. Microsurgical training is predominantly found in the workplace and through face-to-face courses, which have finite access and often high financial incurrence. However, the development of these skills is critical for developing higher levels of skill acquisition in the learner's chosen specialty. Sporadic exposure to microsurgery and inconsistent practice results in poorer skill acquisition, ultimately increasing the time taken to achieve proficiency and maintain skill. Therefore, providing accessible and cost-efficient microsurgical training outside of the operating theater is essential for effective training of junior surgeons in microsurgery. 2 Reduced opportunities have been further exacerbated by the current global climate, with surgical trainees subject to redeployment, reduced face-to-face training opportunities, and restricted theater time.

Surgical education has seen a transition in the delivery of training, with incorporation of online platforms and remote learning to tackle these issues. Simulation training can increase accessibility, reduce cost implications, and provide a constructive learning environment, while reducing patient risk. 3 Microsurgical training is challenging to deliver and assess in this modality, with provision of equipment being the biggest obstacle.

A systematic review conducted by Chen et al identified 24 publications on alternative microsurgery training models using digital magnification tools including smartphone, virtual reality simulators and tablets with models assessed using workshops with trainees or attendings, surveys to end-users, and single-user training to determine users-reported satisfaction and surgical performance. 4 5 6 The application of cameras on smartphones and tablets as an alternative to a surgical microscope has been assessed in several articles. Unfortunately, inconsistency and limitation in magnification, focus, and image quality were identified across multiple hardware, limiting the standardization of training due to variability in lens quality. 7 Alternatively, digital microscopes have been shown to have the potential to be a low-cost tool for trainees to develop skills in microsurgery and develop transferable skills that can be used in the operating room. 8 9

This study aims to compare commercially available digital microscopes, alongside standard binocular surgical microscope performance, and determine whether it can be used as an alternative tool for remote microsurgery simulation.

## Methods

### Microscopes

**Fig. 1:**
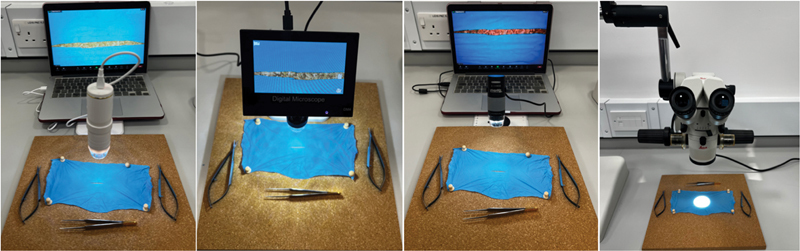
An example set up of the digital microscopes (Left to Right: Pancellant Digital Microscope [PDM], KKnoon Digital Microscope [KDM], Plugable USB Digital Microscope [PLDM], and Leica Table Microscope [LTM]).

A total of four bench microscopes and one smartphone are included in this review. All commercial microscopes were available through accessible online purchasing platforms such as Amazon or a dedicated manufacturer website. 10 Our study includes the iPhone 14 Pro (Smartphone), Pancellant Digital Microscope (PDM), KKnoon Digital Microscope (KDM), Plugable USB Digital Microscope (PLDM), and a Leica binocular microsurgical table microscope (LTM)( Fig. 1 ). 11 12 13 These were chosen as they are felt to be a good representation of commercially available microscopes suitable for surgical training.

### Outcome Measures

Data were collected for cost at the time of purchase, delivery time, image quality, display, and compatibility with devices (Microsoft and Mac) and online platform screen sharing. A subjective questionnaire was distributed to medical students, surgical trainees, and consultants during the period of December 2021 to February 2022. This survey consisted of 11 criteria to assess the suitability for microsurgical training, and outcomes were graded using a 1 to 5 scale ( Appendix A ). Participants were asked to perform interrupted sutures and end-to-end anastomosis on a synthetic model prior to grading each criterion with one equating to poor microscope performance and five equating to the excellent training substitute product. 8–0 sutures, and identical microsurgical needle holders, forceps, and scissors were provided. In accordance with national guidance in the United Kingdom, this study does not require ethical approval.

### Data Synthesis

Data were tabulated for all outcome measures and the mean, mode, and standard deviation calculated. The Kruskal–Wallis test was used to compare the performance of digital microscopes to the standard binocular microscope. This test was performed using Prism Graph Pad statistical software. 14

## Results

A comparison of product characteristics is shown in Table 1 . All digital microscopes were purchased from an online distributor and were received within 3 days or less. The binocular microscope was provided by the Plastic Surgery Department, Royal Free Hospital. Digital microscopes ranged from £26–£56, while costs for binocular training microscopes begin around £300 and smartphone costs begin around £650. All digital microscopes contained simple instructions for setting up the device and did not require installation of software to operate.

**Table 1:** Characteristics of Microscopes 2022. (Pancellant Digital Microscope [PDM], KKnoon Digital Microscope [KDM], Plugable Digital Microscope [PLDM], Leica Table Microscope [LTM], and iPhone 14 Pro [Smartphone])

Microscope	Dimensions cm (L x W x H)	Cost a	Maximum magnification b	Illumination	Display	Compatible with online platform
PDM	18.8 × 11.8 x 4.8	£26.92	50x–1,000x	LED	Laptop	Yes
KDM	15.8 × 11.9 × 21.2	£55.99	1x–1,000x	LED	Device	No
PLDM	8.9 × 3.2 × 3.2	£37.95	1x–250x	LED	Laptop	Yes
LTM	70 × 35 × 40	£300–£2,500 a	6.4/10/16/25/40x	LED	Device	No
Smartphone	14.7 × 7.1 × 0.7	£650–1000	3x (optical) –15x	None	Device	Yes

Abbreviation: LED, light-emitting diode.

a Cost of our model was not available therefore the range of cost of binocular surgical training microscopes is provided.

b Magnification as specified by the manufacturer.

PDM and PLDM rely on displays through a computer/laptop device and are compatible with Microsoft and Mac. They also allowed for screen sharing through online platforms such as Zoom Video Communications, Inc. 15

The KDM display was shown on the device itself, and while recordings could be taken and stored with an SD card, it was not compatible with laptop displays and therefore online platforms in real time. The smartphone display is on the device itself and was found to be compatible with Microsoft teams, however not with Zoom Video Communications, Inc; this was due to the inability to use the main camera, rather than the front camera with this platform. 15

**Fig. 2:**
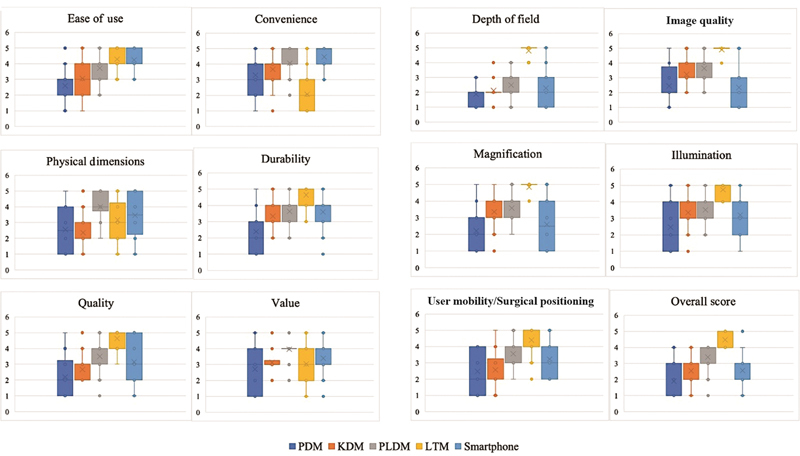
A comparison of outcomes for each microscope from the questionnaire (Box–Whisker plots demonstrate mean, interquartile ranges, standard deviation, and outliers).

The questionnaire was completed by two consultants, nine surgical registrars, fourteen junior trainees, and six medical students giving a total 31 responses. A comparison of each criteria is shown in Fig. 2 .

A comparison of the digital microscopes for each criterion in the survey is shown in Table 2 .

**Table 2:** A comparison of digital microscope outcomes to a binocular table microscope, using the Kruskal–Wallis test, collected at Royal Free Hospital, 2022. (Pancellant Digital Microscope [PDM], KKnoon Digital Microscope [KDM], Plugable Digital Microscope [PLDM], Leica Table Microscope [LTM], and iPhone 14 Pro [Smartphone])

Dunn's multiple comparisons test	Mean rank difference	Significant (Yes/No)	Summary	Adjusted *p* -value
Ease of use/setup				
LTM vs. PDM	63.26	Yes	****	<0.0001
LTM vs. KDM	50.03	Yes	****	<0.0001
LTM vs. PLDM	24.00	No	ns	0.1160
LTM vs. Smartphone	2.604	No	ns	>0.9999
Depth of field				
LTM vs. PDM	85.24	Yes	****	<0.0001
LTM vs. KDM	75.56	Yes	****	<0.0001
LTM vs. PLDM	61.79	Yes	****	<0.0001
LTM vs. Smartphone	74.11	Yes	****	<0.0001
Image quality				
LTM vs. PDM	83.08	Yes	****	<0.0001
LTM vs. KDM	58.39	Yes	****	<0.0001
LTM vs. PLDM	44.29	Yes	***	0.0003
LTM vs. Smartphone	84.45	Yes	****	<0.0001
Magnification				
LTM vs. PDM	88.73	Yes	****	<0.0001
LTM vs. KDM	56.03	Yes	****	<0.0001
LTM vs. PLDM	48.84	Yes	****	<0.0001
LTM vs. Smartphone	77.49	Yes	****	<0.0001
Illumination				
LTM vs. PDM	82.01	Yes	****	<0.0001
LTM vs. KDM	56.66	Yes	****	<0.0001
LTM vs. PLDM	50.52	Yes	****	<0.0001
LTM vs. Smartphone	61.61	Yes	****	<0.0001
Convenience				
LTM vs. PDM	−31.81	Yes	*	0.0153
LTM vs. KDM	−43.87	Yes	***	0.0003
LTM vs. PLDM	−60.24	Yes	****	<0.0001
LTM vs. Smartphone	−77.83	Yes	****	<0.0001
Dimensions				
LTM vs. PDM	18.92	No	ns	0.3506
LTM vs. KDM	26.80	No	ns	0.0690
LTM vs. PLDM	−28.30	Yes	*	0.0477
LTM vs. Smartphone	−10.38	No	ns	>0.9999
Surgeon ease of use/positioning				
LTM vs. PDM	66.88	Yes	****	<0.0001
LTM vs. KDM	65.40	Yes	****	<0.0001
LTM vs. PLDM	31.28	Yes	*	0.0185
LTM vs. Smartphone	42.92	Yes	***	0.0004
Quality				
LTM vs. PDM	78.10	Yes	****	<0.0001
LTM vs. KDM	65.75	Yes	****	<0.0001
LTM vs. PLDM	38.65	Yes	**	0.0020
LTM vs. Smartphone	47.64	Yes	****	<0.0001
Durability				
LTM vs. PDM	83.70	Yes	****	<0.0001
LTM vs. KDM	55.15	Yes	****	<0.0001
LTM vs. PLDM	41.40	Yes	***	0.0007
LTM vs. Smartphone	42.36	Yes	***	0.0004
Value				
LTM vs. PDM	10.93	No	ns	>0.9999
LTM vs. KDM	1.133	No	ns	>0.9999
LTM vs. PLDM	−37.14	Yes	**	0.0026
LTM vs. Smartphone	−14.37	No	ns	0.7355
Overall score				
LTM vs. PDM	86.67	Yes	****	<0.0001
LTM vs. KDM	66.68	Yes	****	<0.0001
LTM vs. PLDM	36.23	Yes	**	0.0046
LTM vs. Smartphone	66.25	Yes	****	<0.0001

Abbreviation: ns, nonsignificant.

Overall, the binocular table microscope performed better in most domains reviewed through the questionnaire.

PLDM had the best overall performance in comparison to the other digital microscopes and performed well across domains. The smartphone was statistically comparable to the PLDM in terms of ease of use, and convenience. Ease of use of the PLDM and smartphone were found to be statistically comparable to the LTM and were deemed valuable training tools by the participants with physical dimensions suitable for practice, scoring better than the LTM in these domains. All digital microscopes were found to be more convenient for trainees with the highest scores demonstrated for the smartphone.

Image quality was reported to be better for the PLDM, and although magnification is superior for the binocular table microscope, the PLDM received the best score for magnification in comparison to the digital microscopes and smartphones.

The PDM was consistently the poorest performing digital microscope. Depth of field was found to be poorer in all digital microscopes with the worst outcomes found for PDM. Trainees commented on the shallow depth considering it detrimental to surgical practice. While the PLDM did not perform as well as the LTM in this domain, trainees commented on the better depth of field in comparison to PDM and KDM and were able to perform the required tasks.

The physical dimension was found to be unsatisfactory for the PDM. Reasons for low scores given to the KDM and PDM for this domain included the height of the microscope being too low to permit adequate performance of the skill assigned (KDM) and instability of the microscope (PDM). One participant also commented on the poor image quality of the PDM and found a lag between their actions and their display.

## Discussion

Simulation training is an important aspect of medical education and provides an opportunity for skill development outside of the workplace, while minimizing risk to patients. Digital microscopes are an alternative tool which could be employed to increase accessibility for junior trainees within the field of microsurgery. They are easily distributable among trainees and are a cost-effective solution which can bridge the gap between initial skill acquisition and performance in the workplace. Remote learning has become an increasingly prevalent modality throughout surgical education; therefore, compatibility with online platforms increases the scope for real-time education and assessment.

Table binocular microscopes represent an optimal simulation of microsurgical practice. However, with limited access to trainees, significant costs, and inability to interact with electronic devices, they are limited in their provision of simulation for juniors and may not always offer a practical solution to independent learning. 7 Additionally, training binocular microscopes may provide only one view of the surgical field, used by the trainee. Assessment of performance can be conducted by reviewing the end-product following completion of the task; however, feedback may be limited for the trainee regarding instrument handling, tissue handling, and efficiency of movement during the task. Increasing commercial binocular training microscopes are incorporating digital displays for recording and demonstration, however, are available at a significantly higher cost and less portable than smaller “pocket” digital microscopes.

The PDM and the PLDM were compatible with displays on electronic devices and could be used with Windows and Mac operating system with no specialist software requirements. The display was easily shared through Zoom Video Communications, Inc., and could be used to provide real-time feedback and assessment of learners. 15 Similarly, the smartphone could provide real-time feedback through Microsoft Teams. Compatibility with electronic devices increases potential opportunities in national and international remote surgical education.

The position of the surgeon will consequentially differ, in comparison to intraoperative use of a binocular scope, with the learner focusing on the display directly in front of them. However, despite this difference, overall learner experience resulting from the survey found that tasks could be performed, with the image quality, magnification and illumination provided by the PLDM.

The image quality of the LTM was superior to all digital microscopes. Visualization of the surgical field is facilitated by the binocular setup; this provides stereoscopic imaging and produces depth perception in real time. 1 In contrast, digital microscopes rely on a digital image to portray the surgical field and, thus, cannot achieve the same experience.

Feedback for user-friendliness and convenience of the smartphone were positive. Students were able to complete suturing tasks and however, found it to be more difficult and challenging. While the image was considered adequate, surgical positioning and depth of field were both highlighted as impeding factors to realistic training. Four participants felt the image was pixelated with magnification. Furthermore, the cost (equal to or more than the LTM), quality, and magnification provided by different models of smartphones could make it challenging to standardize training tools within future courses.

The PDM received the poorest feedback in the participant survey. Although the setup was simple, it was found to have a relatively low image quality and participants commented on the quality of the scope, finding its instability detrimental to surgical practice. In contrast, results for the PLDM were higher in comparison to its digital counterparts. The quality of the scope, image quality, magnification, and efficacy in relation to surgical skill performance, highlighted as superior in comparison. Surgical trainees were able to perform the task allocated with sufficient visualization of the surgical field.

While the KDM was found to have high image quality and magnification, trainees commented on the dimensions and found that the height of the microscope interfered with their performance of interrupted sutures. Another limitation of this scope is the inability for the display to be shared through online platforms, therefore limiting its applicability in teaching and training.

Depth of field remains a challenging aspect to achieve through the employment of digital microscopes with all having a relatively shallow view in comparison to the binocular microscope. While they did not perform as well in this domain, the PLDM received the highest score within the commercially available digital scopes and the participants were still able to perform the surgical task with some adjustments. However, with the digital view, there is an inability of all digital microscopes to provide depth perception, and therefore, the variation in score by participants in this domain could be related to the image quality.

Chai et al also highlighted the shallow depth of field experienced with digital microscopes and, however, found that the microscope allowed for cleaning the adventitia and suturing of the back wall for microvascular anastomosis. 7 Overall, participants found the digital microscope to be a useful tool in developing proficiency and skill acquisition in this field, with convenience being a contributing factor to continuous training; however, the benefit may plateau for more advanced practitioners.

The digital microscopes and smartphones chosen as part of this review were included because of their cost, availability, and reviews of their performance at the time of this study. However, given the fluctuation within digital technologies, there may be both better and worse tools available in the market.

## Conclusion

While a table binocular microscope is considered the gold standard tool for training in this field, digital microscopes can provide an alternative training tool for microsurgical simulation of junior trainees. While shallow depth perception remains a challenging aspect to simulate, the PLDM was found to be a cost-effective tool which can facilitate microsurgical practice, good overall participant experience in comparison to other digital microscopes in this study, and satisfactory visualization, magnification, and illumination to facilitate microsurgical skill acquisition.

## Appendices

**Appendix A:** Questionnaire for comparison of microscopes in surgical simulation applications

Equipment specific criteria and description	
Ease of use and device setup	How easy is the microscope to set up including focus, magnification, and illumination?
Convenience	Is the microscope accessible, quick to use independently, transportability?
Physical dimensions	Is the size of the microscope compatible with microsurgical use?
Durability	How sturdy is the microscope?
Quality	Is the microscope of satisfactory quality for use in surgical education?
Value	Does the price and quality of this microscope meet your expectations relative to microsurgical training?
Microsurgical application outcomes	
Depth of surgical field	Is the depth of the surgical field perceived as shallow/poor or satisfactory?
Image quality	Can the trainee visualize the surgical field clearly and identify structures?
Magnification	Does the microscope provide suitable magnification for the performance of microsurgical tasks?
Illumination	Is the light strong enough to visualize the field? Is there interference from ambient lighting?
Surgical positioning/mobility	How does the microscope affect the positioning of the surgeon and the efficacy of movement during the task?

Note: Grading scale: 1 = poor; 2 = unsatisfactory; 3 = satisfactory; 4 = good; 5 = excellent.
